# Amino Acids during Pregnancy and Offspring Cardiovascular–Kidney–Metabolic Health

**DOI:** 10.3390/nu16091263

**Published:** 2024-04-24

**Authors:** You-Lin Tain, Chien-Ning Hsu

**Affiliations:** 1 Division of Pediatric Nephrology, Kaohsiung Chang Gung Memorial Hospital, Kaohsiung 833, Taiwan; tainyl@cgmh.org.tw; 2 College of Medicine, Chang Gung University, Taoyuan 333, Taiwan; 3 Institute for Translational Research in Biomedicine, Kaohsiung Chang Gung Memorial Hospital, Kaohsiung 833, Taiwan; 4 Department of Pharmacy, Kaohsiung Chang Gung Memorial Hospital, Kaohsiung 833, Taiwan; 5 School of Pharmacy, Kaohsiung Medical University, Kaohsiung 807, Taiwan

**Keywords:** amino acid, cardiovascular disease, chronic kidney disease, metabolic syndrome, hypertension, developmental origins of health and disease (DOHaD), pregnancy

## Abstract

Amino acids are essential for normal pregnancy and fetal development. Disruptions in maternal amino acid metabolism have been associated with various adult diseases later in life, a phenomenon referred to as the developmental origins of health and disease (DOHaD). In this review, we examine the recent evidence highlighting the significant impact of amino acids on fetal programming, their influence on the modulation of gut microbiota, and their repercussions on offspring outcomes, particularly in the context of cardiovascular–kidney–metabolic (CKM) syndrome. Furthermore, we delve into experimental studies that have unveiled the protective effects of therapies targeting amino acids. These interventions have demonstrated the potential to reprogram traits associated with CKM in offspring. The discussion encompasses the challenges of translating the findings from animal studies to clinical applications, emphasizing the complexity of this process. Additionally, we propose potential solutions to overcome these challenges. Ultimately, as we move forward, future research endeavors should aim to pinpoint the most effective amino-acid-targeted therapies, determining the optimal dosage and mode of administration. This exploration is essential for maximizing the reprogramming effects, ultimately contributing to the enhancement of cardiovascular–kidney–metabolic health in offspring.

## 1. Introduction

Appropriate morphology and the normal functional development of the cardiovascular system, kidneys, metabolic organs, and other tissues are crucial for fetal growth and development [1]. Maternal nutrition must be adequate during pregnancy in order to accommodate placental formation and support fetal development [2,3]. Imbalances in maternal nutrition have been associated with the development of many adult diseases later in life [4,5,6,7]. Recognized globally as the notion of the developmental origins of health and disease (DOHaD) [8,9], this idea has garnered a widespread consensus. In contrast, an increasing body of evidence indicates that intervening during the early stages of developmental plasticity can improve, or even reverse, the negative effects linked to developmental programming through a process of reprogramming [10,11]. Placing increased emphasis on the application of nutritional interventions for reprogramming strategies, recent research studies have begun to address the prevention of disorders associated with DOHaD, including cardiovascular–kidney–metabolic (CKM) syndrome [7,12,13].

Characterized as a systemic disorder within its first definition in a new American Heart Association scientific statement [14], CKM syndrome involves pathophysiological interconnections among metabolic risk factors like obesity and diabetes, kidney disease, and diseases impacting the cardiovascular system. This intricate interplay leads to multiorgan dysfunction and an elevated risk of adverse cardiovascular and kidney outcomes. Comprising four well-defined stages from stage 0 to stage 4, CKM syndrome exhibits a classification system wherein different key constituents manifest at various stages, contributing to the diverse progression and severity observed within the intricate spectrum of CKM syndrome [14,15]. Despite the recommendation for a holistic approach to manage the entire syndrome, rather than concentrating on individual diseases, there is still a lack of comprehensive therapeutic guidelines [15]. Recognizing the impact of maternal nutrition on offspring health and disease, prioritizing early nutritional interventions holds the potential to alleviate the future burden of CKM syndrome.

Amino acids assume a pivotal role in diverse physiological functions within the human body. Of particular significance is the existence of essential amino acids, which the body is unable to produce independently and must acquire through dietary intake. Consequently, ensuring a sufficient supply of amino acids during all trimesters is imperative for promoting normal pregnancy and fostering optimal fetal development [16,17]. While guidelines for protein intake during pregnancy are articulated through recommended dietary allowance (RDA) values and estimated average requirement (EAR) [18], the lack of specific recommendations for individual amino acids in the context of pregnant women is worth noting [16]. Despite indications from human and animal studies suggesting that certain amino acid supplementation during prenatal stages could be a promising approach to enhance healthy fetal growth [19], there is limited knowledge regarding their reprogramming effects on offspring with CKM syndrome.

Recent studies have honed in on the impact of the gut microbiome in CKM traits [20,21,22]. As nutrients interact with gut microbes, crucial secondary metabolites are released, which are subsequently absorbed by the host. Various proposed mechanisms connect the gut microbiota and derived metabolites to CKM syndrome, including alterations in the gut microbiome, the dysregulation of short-chain fatty acids (SCFA), increases in trimethylamine-*N*-oxide (TMAO), and microbiota-derived uremic toxins [23,24,25,26]. Maternal nutrition has demonstrated the capacity to alter the balance of the gut microbiome, implicating offspring health and disease [27]. However, there is limited information available on whether and how maternal amino acid supplementation might impact the gut microbiota, potentially playing a role in programming and reprogramming CKM syndrome in adult offspring.

## 2. Materials and Methods

### 2.1. Data Sources and Search Strategy

**Figure 1:**
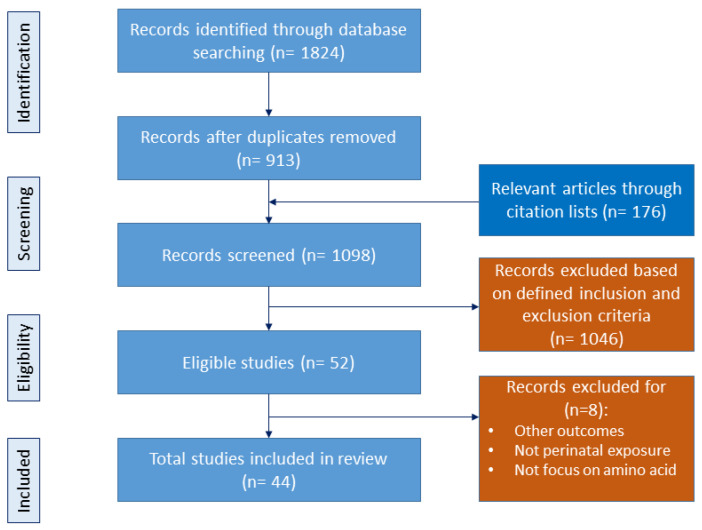
Flow diagram of the literature search and selection.

The objective of this review is to consolidate recent findings and underscore the impact of amino acids during pregnancy on fetal programming, the modulation of the gut microbiota, and the intricate interplay among these elements in the developmental programming of CKM syndrome. We adhered to the preferred reporting items for systematic reviews and meta-analyses (PRISMA) guidelines throughout our review process. The study selection process is documented in Figure 1.

To compile a comprehensive literature review, relevant studies published in English were identified through a search of the MEDLINE, Embase, and Cochrane Library databases. The search employed pertinent keywords related to DOHaD, amino acids, gut microbiota, and CKM syndrome. The utilized search terms comprised the following: “obesity”, “diabetes”, “metabolic syndrome”, “dyslipidemia”, “insulin resistance”, “hyperglycemia”, “liver steatosis”, “kidney disease”, “cardiovascular disease”, “hypertension”, “atherosclerosis”, “heart failure”, “cardiorenal syndrome”, “developmental programming”, “DOHaD”, “offspring”, “progeny”, “mother”, “prenatal”, “pregnancy”, “reprogramming”, “gut microbiota”, “short-chain fatty acid”, “trimethylamine-*N*-oxide”, “uremic toxin”, and “amino acid”.

### 2.2. Eligibility Criteria

Peer-reviewed studies fulfilled the search terms that were involved. Our inclusion criteria encompassed studies published from January 2000 to January 2024, written in English. We broadened our scope to include epidemiological investigations, clinical trials, and animal models. Editorials, conference abstracts, letters, notes, and comments were excluded. Additionally, we scrutinized the reference lists for supplementary relevant sources.

### 2.3. Data Extraction and Synthesis

Initially, we conducted comprehensive searches across various databases using specific search terms, resulting in the retrieval of 1824 articles. All duplicate papers were double checked and excluded. Additionally, we identified 176 relevant articles through citation lists. From these combined sources, a total of 1098 studies were screened for inclusion based on the predefined criteria. A total of 52 articles remained eligible for inclusion in this study. Subsequently, a secondary manual screening was performed, resulting in the exclusion of studies that did not meet the inclusion criteria. Following this process, 44 studies remained for inclusion in our review.

## 3. Impact of Amino Acids on Pregnancy and Fetal Development

### 3.1. The Impact of Amino Acids on Pregnancy

As pregnancy progresses, there is typically an increase in the overall concentration of amino acids, which is, at least in part, due to the higher demand for protein synthesis. The current RDA for protein, part of the Dietary Reference Intake (DRI), is set at 1.1 g/kg/day for pregnant individuals. This represents a higher value compared to the non-pregnant state, where the RDA is set at 0.8 g/kg/day [18]. Hypoaminoacidemia during fasting is associated with pregnancy, a phenomenon that is evident in the early stages of gestation and continues throughout the entire pregnancy [28,29]. Notably, there is a more pronounced decline in glucogenic amino acids, including serine, alanine, glutamine, threonine, and glutamate, during pregnancy, particularly in the early stages [28]. While it is commonly asserted that the amino acid levels should rise proportionally with increased protein requirements during pregnancy, the specific amino acid needs in human pregnancy are scarcely reported [16]. For instance, a 27% rise in lysine requirements has been reported during late pregnancy compared to that of early pregnancy [30]. Similarly, during late gestation in human pregnancy, there may be a 40% higher demand for phenylalanine compared to that of early gestation [31]. Swine models demonstrate elevated requirements for threonine (55%), lysine (45%), isoleucine (63%), and tryptophan (35%) in late pregnancy, as opposed to those of the early stages [16]. The adaptive increase in isoleucine, a branched-chain amino acid (BCAA) serving as the primary nitrogen source for ureogenic amino acids, is hypothesized to target the overall nitrogen conservation and heightened protein synthesis. However, it remains unclear whether other BCAA requirements also increase and the precise mechanism behind this adaptation. These discoveries indicate possible implications for dietary amino acid recommendations specific to the gestational stages.

### 3.2. The Impact of Amino Acids on Fetal Development

**Figure 2:**
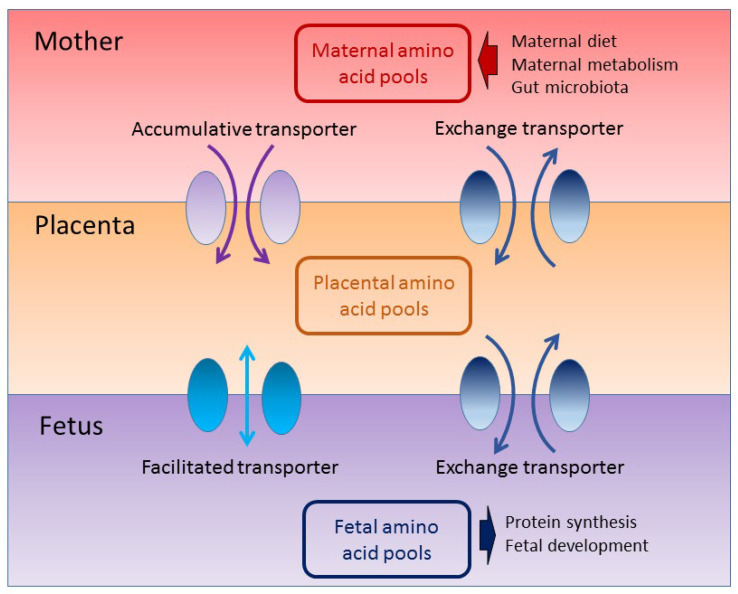
A cartoon showing placental amino acid transporters that determine the amino acid pool between the mother, placenta, and fetus. Flow diagram of the literature search and selection.

The amino acid levels are higher in fetal circulation as a result of active transport mechanisms across the placenta and are essential to afford the essential building blocks for protein synthesis and cellular development [32,33]. Influencing the pool of amino acids accessible for transport to the fetal circulation is an active role played by the placenta [34]. The placenta features the following three distinct types of amino acid transport systems: accumulative, exchange, and facilitated transporters [34] (Figure 2).

Facilitating the transfer of amino acids in the placenta is predominantly governed by nutrient-sensing signaling, including the mechanistic target of the rapamycin (mTOR) pathway [35]. Studies have previously indicated reduced placental amino acid transfer, mTOR activity, and activity of amino acid transporters in cases of intrauterine growth retardation (IUGR) [36,37,38]. Furthermore, the substantial reduction in the activity of system A, system L, and taurine amino acid transporters is a notable consequence of mTOR inhibition induced by rapamycin [38]. These findings highlight the placenta’s precise regulation of amino acid concentrations in fetal circulation, a critical factor for ensuring normal fetal development.

Total amino acid levels have shown associations with fetal outcomes, particularly infant birth weight. In a prior investigation, positive correlations were observed among the concentrations of ornithine, serine, lysine, arginine, proline, and neonatal birth weight [39]. Arginine, acting as a shared substrate for both nitric oxide (NO) and polyamines, plays a crucial role in fetal development and placental angiogenesis [40], illustrating the significance of this association. In contrast, serine is not significantly transported to the fetus [41]. Therefore, these correlations do not necessarily imply key roles for these amino acids in fetal growth. Considering the fact that alterations in a specific amino acid may influence the metabolic processes of others, further studies have shifted their focus to exploring both individual amino acids and the equilibrium of the amino acid pool in fetal development. Urgent exploration is warranted to delve deeper into these aspects.

The maternal low-protein diet serves as a commonly employed experimental model for investigating the effects of early nutrition on the adult offspring’s health [42]. Adult rat offspring born to dams fed low-protein diets displayed a reduced body weight, elevated blood pressure (BP), and metabolic abnormalities [42]. Nevertheless, epidemiological research on the outcomes of either insufficient or excessive intake of specific amino acids on fetal development and subsequent outcomes in the offspring is currently insufficient.

## 4. The Connection between Dietary Amino Acids and Gut Microbiota

Currently, the proposed mechanisms behind nutritional programming include epigenetic regulation, dysregulated nutrient sensing, glucocorticoid programming, the aberrant renin–angiotensin system (RAS), oxidative stress, and dysbiotic gut microbiota [5,6,7,43,44,45]. Among them, gut microbiota stands out as the pivotal mechanism connecting amino acids to the developmental programming of CKM syndrome.

Within the human gut, a multitude of microbial species, numbering in the thousands, collectively constitute the gut microbiota. This vast microbial community exerts considerable influence over the uptake, processing, and retention of dietary nutrients, thereby significantly shaping host’s physiology [46]. The gut microbiota holds a crucial role in governing the digestion and absorption of amino acids. It is essential to differentiate between the total amino acid reservoir and the amino acid composition, which examines the specific distribution of individual amino acids. The presence of resident bacterial species in the gut intricately influences the distribution of free amino acids within the gut [47].

Examining the resident bacterial species within the human colon has revealed significant findings concerning substantial populations of bacteria adept in the fermentation of proteins and amino acids [48]. Specifically, the key drivers of amino acid fermentation in the large intestine are bacteria belonging to the *Clostridium* genus, which are particularly crucial for proline or lysine use. Additionally, the genus *Peptostreptococcus* plays a pivotal role in the utilization of tryptophan or glutamate. It is crucial to acknowledge that various species may assume prominent roles in amino acid metabolism within the large intestine, including bacteria from the genera *Bacteroides*, *Fusobacterium*, and *Veillonella*, as well as the species *Selenomonas ruminantium* and *Megasphaera elsdenii*. Apart from consuming amino acids, the gut microbiota plays a crucial role in the generation of amino acids, encompassing de novo biosynthesis. Various species, including *Streptococcus bovis*, *Selenomonas ruminantium*, and *Prevotella bryantii*, have been identified as actively participating in the de novo synthesis of amino acids [49].

SCFAs are important microbial-derived metabolites formed during the bacterial fermentation of carbohydrates, mainly consisting of acetate, propionate, and butyrate. In the gut, microbial protein fermentation yields numerous amino acids that serve as synthetic precursors to SCFAs [50,51]. Certain anaerobic bacteria have the capability to metabolize specific amino acids to produce acetate, and this group includes glycine, threonine, glutamate, and ornithine [51]. Additionally, threonine, lysine, and glutamate can contribute to the synthesis of butyrate. Another SCFA, propionate, is primarily derived from the metabolism of threonine [52]. These findings underscore the remarkable adaptability of threonine among the amino acids used for SCFA synthesis, as it contributes to the production of each of the three fundamental SCFAs. In light of these findings, it is paramount to acknowledge that the intake and synthesis of amino acids by the gut microbiota play a significant role in shaping the amino acid reservoir. Additionally, amino acids can undergo catabolism through distinct pathways in the gut. The diverse characteristics of amino acid catabolism within the indigenous species of the gut microbiota have the potential to generate both favorable and unfavorable effects on the host [53].

## 5. Amino Acids and CKM Syndrome

Apart from serving as the building blocks for proteins, amino acids play crucial roles in the essential pathways that regulate cell growth, metabolism, biosynthesis, neurotic transmission, and immunity [54]. Disturbances in the amino acid metabolism have been associated with various pathological conditions [54], containing significant components of CKM syndrome [14,15]. The subsequent sections delve into each of these aspects in detail.

### 5.1. Hypertension and Cardiovascular Disease

Numerous amino acids have roles in the regulation of BP. As an example, intracisternal injections of serine, alanine, taurine, and glycine in conscious rats result in depressor responses, while arginine, proline, glutamate, cysteine, aspartic acid, and asparagine lead to pressor responses [55]. In blood vessels, arginine, homocysteine, branched-chain amino acids (BCAAs), and tryptophan are known to influence the development of atherosclerosis [56]. While there is an identified increased risk of CVD in pregnant women associated with inadequate levels of certain amino acids like alanine and glycine [57], there is still a lack of specific recommendations regarding individual amino acids and their precise dosage requirements for pregnant women. Several arginine-related amino acids may contribute to NO bioavailability, including arginine, methylated arginine, citrulline, and its homolog L-homoarginine. Arginine serves as a precursor for NO, a key player in endothelium-dependent vasodilation within the blood vessels [58]. Arginine has the potential to undergo methylation, resulting in either monomethylated arginine or dimethylated arginine. Asymmetric dimethylarginine (ADMA), an endogenous inhibitor of nitric oxide synthase (NOS), holds the ability to markedly diminish NO production, thereby contributing to the development of CVD [59]. Patients with hypercholesterolemia, coronary artery disease, and peripheral vascular disease usually have raised ADMA [60,61].

The synthesis of arginine faces challenges in situations where there is a decline in the functioning of the small intestine and kidneys, resulting in a dietary need for arginine. Citrulline is a precursor for de novo arginine synthesis. The addition of citrulline can increase renal NO production and prevent hypertension in spontaneously hypertensive rats (SHRs) [62]. Homoarginine, a nonproteinogenic amino acid structurally similar to arginine, has been reported to be a substrate in NO synthesis, akin to arginine [63]. In rat models of heart failure, the administration of homoarginine demonstrates a capacity to enhance cardiac function and mitigate remodeling in response to pressure overload [64]. Methionine, homocysteine, cysteine, and taurine are the four common sulfur-containing amino acids. Homocysteine is a sulfur-containing amino acid formed during the metabolism of methionine, an essential amino acid obtained from dietary sources. Elevated levels of homocysteine have been associated with an increased risk of CVD [65]. Hyperhomocysteinemia may stimulate ADMA production, damage endothelial function, elevate BP, and cause atherosclerosis [65,66]. Another sulfur-containing amino acid, cysteine, functions as a precursor for hydrogen sulfide (H_2_S) and is an integral component of glutathione, which is a crucial antioxidant. Due to the interconnected roles of glutathione and H_2_S signaling in BP regulation [67,68], cysteine is recognized for its potential antihypertensive effects [69]. In a comparable manner, taurine, another amino acid containing sulfur, exhibits vasodilatory effects [70]. Numerous studies, as outlined in reviews elsewhere [71], have explored the potential antihypertensive benefits of taurine supplementation in diverse hypertensive rat models. Likewise, the BCAAs—leucine, isoleucine, and valine—are related to CVD [72]. The essential amino acids known as BCAAs are primarily obtained from the diet, even if they can also be synthesized by intestinal microbes [72].

Elevations in plasma BCAA levels, particularly isoleucine, are associated with hypertension and CVD in numerous epidemiological cohorts, as reviewed elsewhere [73]. Furthermore, tryptophan and its metabolites have been linked to atherosclerosis and hypertension [74,75]. Tryptophan, an essential amino acid necessitating dietary intake, undergoes metabolism through three primary pathways in the gut, as follows: (1) the kynurenine pathway, active in both immune and epithelial cells; (2) the indole pathway, facilitated by the gut microbiota; and (3) the serotonin pathway, working in enterochromaffin cells [76]. The kynurenine pathway accounts for over 95% of the absorbed tryptophan catabolism, with only 1–2% and 2–3% of dietary tryptophan being transformed into the serotonin and indole pathways, respectively [77,78]. Despite the vasodilatory properties of tryptophan [79], the activation of the kynurenine pathway has been linked to hypertension [80]. In patients with CKD, gut-microbiota-produced uremic toxins derived from tryptophan, primarily through the indole and kynurenine pathways, are associated with CVD [81,82]. The role of serotonin in the control of BP is intricate and remains unclear [83]. While serotonin induces acute arterial constriction [83], prolonged serotonin administration leads to a sustained decrease in BP [84]. These findings indicate that disruptions in amino acid metabolism could potentially play a role in the development of hypertension and CVD. Nevertheless, the precise mechanisms and interplay among the amino acids remain subjects of ongoing research, and the individual responses to these factors may differ.

### 5.2. Obesity

The amino acid metabolism contributes to the overall energy balance in the body, and an imbalance in energy homeostasis is implicated in the onset of obesity. Certain amino acids, like BCAAs, methionine, tryptophan and its metabolites, and glutamate have been studied for their potential role in promoting muscle protein synthesis and maintaining lean body mass [85]. The plasma levels of BCAAs are increased in patients with obesity [86]. A growing body of evidence indicates that the supplementation of leucine in the diet has a beneficial impact on parameters related to obesity [87]. In rodent models of diet-induced obesity, methionine restriction has shown improvements in body weight gain, glucose metabolism, and insulin sensitivity through a communication mechanism between the adipose tissue and the skeletal muscle, involving the release of the adiponectin [88,89]. A recent systematic review focusing on pediatric obesity revealed abnormal levels of several amino acids, notably those belonging to tryptophan metabolism, including the kynurenine pathway [90]. Tryptophan restriction could modulate energy balance and induce weight loss in animal models of obesity [91,92]. Glutamate is another amino acid linked to obesity. Circulating glutamate levels are positively associated with central obesity [93]. However, the underlying pathophysiological pathways responsible for this association are still unclear.

### 5.3. Diabetes

A comprehensive meta-analysis indicated elevated levels of various amino acids, including BCAAs, aromatic amino acids, and glutamine, in individuals with type 2 diabetes compared to their control counterparts [94]. The rise in BCAA levels can be linked to a decline in the flow of metabolic processes through the citric acid cycle within muscle tissues [95]. Experimental studies utilizing rodent models have demonstrated that reducing BCAA levels through a BCAA-restricted diet or by activating the rate-limiting enzyme in BCAA catabolism yields clear beneficial effects on glucose homeostasis [96]. Although high aromatic amino acids levels are attributed to elevated levels of BCAAs, their precise impact on diabetes remains uncertain. Glutamine has emerged as a pivotal amino acid in the ruling of insulin sensitivity and glucose stability. Supplementation with glutamine has been shown to forestall the onset of insulin resistance by mitigating inflammation and fostering insulin sensitivity in skeletal muscle, as evidenced in a mouse model of obesity [97]. Glycine levels exhibit a negative correlation with obesity and insulin resistance in diabetes patients. Evolving evidence suggests that supplementing the diet with glycine increases insulin levels, reduces systemic inflammation, and enhances glucose tolerance in diabetes patients [98]. Nevertheless, the exact role of glycine in glucose regulation, beyond its potential as a biomarker, remains less evident.

### 5.4. NAFLD and Dyslipidemia

Non-alcoholic fatty liver disease (NAFLD) arises as a result of metabolic disorders, encompassing obesity, insulin resistance, and metabolic syndrome. Dyslipidemia is pivotal in the progression of NAFLD. The gathering of free fatty acids and lipid metabolites within hepatocytes disrupts insulin-triggered cell signaling, ultimately initiating the development of NAFLD [99]. In the liver, the amino acid metabolism can impact the synthesis of glutathione, insulin resistance, oxidative stress, and inflammation [100]. In patients with NAFLD, changes in the circulating amino acids can be noted, with increases in BCAAs and aromatic amino acids and decreases in the amino acids associated with glutathione synthesis (glutamine, serine, and glycine) [101]. Increased amino acid availability (e.g., BCAAs and aromatic amino acids) could result in intrahepatic fat accumulation by interfering with fat and glucose metabolism. Prior work indicates that attenuating experimental NAFLD is observed with a glycine-based treatment, which stimulates hepatic fatty acid oxidation and glutathione synthesis [102]. Similarly, glutamine supplementation could reduce oxidative stress in the liver, which was shown to inhibit inflammation and improve hepatic steatosis in a rat model of NAFLD [103]. Although methionine deficiency has been used in a methionine- and choline-deficient diet mouse model to study NAFLD [104], the role of methionine in NAFLD remains less clear.

### 5.5. CKD

The human kidney plays a crucial role in maintaining the homeostasis of amino acid levels within the body. The kidney serves as the primary organ for the elimination of glutamine and proline, as well as the net release of certain amino acids like arginine, tyrosine, and serine, which are newly synthesized within the kidney for export to other tissues [105]. The insufficient production of tyrosine in the kidneys is observed in patients with CKD, potentially resulting in protein depletion and the impaired synthesis of aromatic amine modulators [106]. Furthermore, recent observations suggest that, in patients with CKD, low arginine availability and elevated ADMA are associated with reduced de novo arginine and NO synthesis [107].

Uremic toxins, primarily derived from tryptophan, are not only a consequence of renal dysfunction, but also contributors to the progression of CKD [108]. Indoxyl sulfate (IS) and p-cresyl sulfate (PCS) are well-known uremic toxins originating from tryptophan. In patients with CKD, there is a reduced urinary excretion of various microbial tryptophan metabolites, such as IS and PCS. These tryptophan metabolites serve as ligands for the AHR [109]. The activation of AHR can induce oxidative stress, initiate inflammation, and modulate the Th17 axis, contributing to the progression of CKD [110].

**Figure 3:**
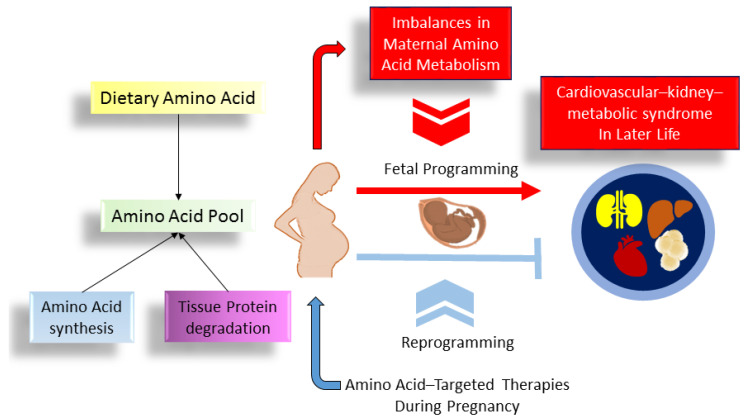
An overview of the role of maternal amino acid metabolism in the developmental programming of offspring cardiovascular–kidney–metabolic (CKM) syndrome.

It is important to highlight that maternal amino acid levels not only influence fetal growth but also impact postnatal growth trajectories [111]. These trajectories are intricately linked to various components of CKM syndrome, such as obesity, diabetes [112], hypertension [113], and cardiovascular disease [114]. As outlined above, there are intricate associations between the imbalances in maternal amino acid metabolism, fetal programming, and CKM syndrome in the later stages of life (Figure 3).

## 6. Effects of Perinatal Amino Acid Supplementation on Offspring CKM Syndrome

Considering the importance of amino acids in fetal programming, the perinatal supplementation of amino acids may be an effective therapeutic option to improve perinatal and long-term offspring health (Figure 1). Currently, several amino-acid-targeted therapies have been examined to improve pregnancy outcomes and fetal growth in both human and experimental research [115].

Forming three intriguing supplementation groups, due to their impact on fetal growth, are the arginine family, BCAAs, and methyl donors. Although a meta-analysis indicated the efficacy of this approach for the arginine family, the difficulty in determining the most efficient amino acids was exacerbated by the limited number of studies conducted in complicated pregnancy settings compared to those in normal growth conditions, particularly concerning BCAAs and methyl donors [115]. Less research has been conducted on perinatal amino acid supplementation in relation to its effects on the offsprings’ long-term outcomes. In the present review, our focus is solely on amino acid supplementation starting during pregnancy and/or lactation as a reprogramming strategy to prevent CKM traits in rodent animal models, as summarized in Table 1 [62,116,117,118,119,120,121,122,123,124,125,126,127,128,129,130,131,132,133,134,135,136,137,138].

Numerous developmental programming models have been investigated, encompassing diverse approaches such as the following: the maternal protein restriction model [116,135], the maternal caloric restriction model [117,136], antenatal dexamethasone exposure [118], streptozotocin (STZ)-induced diabetes [119,126], the maternal N^G^-nitro-L-arginine-methyl-ester (L-NAME) exposure model [120,133], maternal CKD [121,125,130,138], a maternal high-fat/high-fructose diet [122], the genetic hypertension model [62,129,130], a combination of antenatal dexamethasone and a postnatal high-fat diet [131], the suramin-induced preeclampsia model [132], maternal nicotine exposure [134], and a maternal and post-weaning high-fat diet [137]. The primary focus in evaluating the components of CKM syndrome involves hypertension, followed by kidney disease, obesity, diabetes, and dyslipidemia. Reprogramming effects have been observed through amino-acid-targeted therapies in rats ranging from 4 weeks to 50 weeks old, coarsely equivalent to human ages spanning from young children to middle adulthood [139]. Amino acid supplementation utilized as reprogramming interventions includes arginine [116], citrulline [62,117,118,119,120,121], taurine [122,123,124,125,126,127,128,129], cysteine [130,131,132,133,134], glycine [135], BCAAs [136,137], and tryptophan [138]. The subsequent sections will delve into each of these aspects in detail.

### 6.1. Arginine

Studied in human diseases as a method of enhancing NO bioavailability, arginine supplementation, with an oral range of 3–100 g/day, has been investigated [140]. Gastrointestinal disturbances have been documented when single doses exceed 9 g, or when the daily dosing regimen exceeds 30 g [141]. As of now, the outcomes of arginine supplementation in human trials are inconclusive [142]. The protection against hypertension of adult offspring in various genetic hypertensive rat models has been demonstrated in prior work employing perinatal arginine supplementation combined with taurine and antioxidants [143,144,145]. Nonetheless, as indicated in Table 1, the examination of the protective effects of arginine supplementation alone on offspring CKM syndrome is limited to a singular study. This study demonstrated that administering arginine at a daily dose of 200 mg/kg during lactation effectively prevented hepatic insulin signaling and the expression of gluconeogenic enzymes [116]. While post-weaning arginine supplementation alone has been shown to prevent hypertension in offspring rats complicated by maternal caloric restriction or diabetes [146,147], it remains unclear as to whether perinatal arginine supplementation alone is associated with these effects. Furthermore, the protective effects on IUGR in ovine and swine have been demonstrated with arginine supplementation during the gestational period [148,149]. However, the reprogramming actions of arginine therapy during pregnancy, beyond its impact on IUGR, have not been thoroughly examined in these species at present.

### 6.2. Citrulline

Recognized as a supplementary approach to increase plasma arginine levels and boost NO generation, oral citrulline supplementation has garnered interest, due to its capacity to circumvent hepatic metabolism and transform into arginine within the kidneys [150]. In the human context, the safety and tolerability of citrulline supplementation have been demonstrated through the administration of single oral doses ranging from 2 to 15 g [151].

Used as a reprogramming intervention during pregnancy and lactation, citrulline supplementation aims to protect adult offspring against hypertension in various rat models, covering antenatal dexamethasone exposure [118], STZ-induced diabetes [119], the maternal L-NAME exposure model [120], and maternal CKD [121]. A study revealed that, in the offspring of dams with STZ-induced diabetes, where a reduced nephron number and increased ADMA contribute to adult kidney disease and hypertension, citrulline supplementation during pregnancy and lactation prevented these conditions by manipulating the ADMA–NO pathway [119]. In the maternal L-NAME exposure model, where maternal citrulline supplementation was implemented, it successfully prevented offspring hypertension programmed by maternal NO depletion. Linked with over 300 genes, this depletion resulted in a notable modification of the renal transcriptome in adult offspring [120]. These observations indicate that early citrulline supplementation has a lasting influence on kidney development, bringing about alterations in the renal transcriptome. Consequently, further exploration is needed to fully understand the potential implications of epigenetic regulation by citrulline during the initial stages of programming.

### 6.3. Taurine

The most frequently supplemented amino acid during pregnancy is taurine, as indicated in Table 1, which has been extensively studied in various aspects of CKM syndrome. As the most prevalent sulfur-containing amino acid [152], taurine is predominantly acquired through dietary sources, although its synthesis is also possible from cysteine. The table illustrates that maternal taurine supplementation provides protection against hypertension programmed by maternal high-sugar intake [124], maternal CKD [125], STZ-induced diabetes [126], or maternal dyslipidemia [127]. Furthermore, perinatal taurine supplementation has demonstrated efficacy in preventing hypertension in SHRs and stroke-prone spontaneously hypertensive rats (SHRSP) [128,129]. In addition, maternal taurine supplementation can ameliorate obesity programmed by a maternal high-fat/high-fructose diet [122] and maternal dyslipidemia [127]. Using a NOD mice model, perinatal taurine treatment was shown to delay the onset time of diabetes from 18 to 30 weeks in female offspring, and from 30 to 38 weeks in male offspring [123]. With respect to gut microbiota, taurine plays a crucial role in safeguarding the host, acting as a vital energy source for microbes, providing defense against pathogens, and regulating bacterial colonization [153]. In the context of maternal CKD, the protective effects of perinatal taurine treatment on offspring hypertension are intricately linked to alterations in the gut microbiota. This treatment results in an increased abundance of the genera *Asteroleplasma, Bifidobacterium*, and *Dehalobacterium*, coupled with a reduction in *Erisipelactoclostridium* [125]. The restoration of *Bifidobacterium* levels, which were diminished due to maternal CKD, through taurine administration is attributed to its probiotic capability of preventing hypertension [125].

### 6.4. Cysteine

Recognized as a rate-limiting factor in the synthesis of glutathione [69], cysteine plays a crucial role in cellular processes. Experimental studies have employed cysteine supplementation to generate endogenous H_2_S [154]. While early post-weaning cysteine supplementation has been stated to augment the H_2_S signaling pathway and avert hypertension in high-salt-treated SHRs [155], to date, only one study has evaluated the protective actions of gestational cysteine supplementation in a maternal CKD model [130]. The findings of this study revealed that supplementation with either L- or D-cysteine effectively prevented hypertension in offspring primed by maternal CKD [130]. The treatment with L-cysteine shielded the adult offspring from hypertension, promoting increased H_2_S production and enhancing the presence of beneficial genera such as *Oscillibacter* and *Butyricicoccus*. It also resulted in the depletion of indole-producing genera like *Akkermansia* and *Alistipes*, along with a reduction in various indole metabolites.

On the other hand, D-cysteine supplementation results in elevated levels of 3-hydroxykynurenine, kynurenic acid, and xanthurenic acid in the kynurenine pathway. It also decreased serotonin and 5-hydroxytryptophan in the serotonin pathway, while enriching the abundance of genera *Odoribacter* and *Bacteroides*. The gut microbiota’s degradation of cysteine releases H_2_S, which, in turn, influences the composition of the gut microbiota [156], therefore, supporting the idea that amino acids could serve as prebiotics to confer beneficial effects on host health [157]. Cysteine, when absorbed into cells, loses its antioxidant properties. Hence, for this purpose, *N*-acetylcysteine (NAC), which is a stable analogue of cysteine, is frequently employed. Table 1 presents evidence of the antihypertensive actions of perinatal NAC therapy in various animal models, including those involving prenatal dexamethasone treatment and exposure to a postnatal high-fat diet [131]. Additionally, positive outcomes were observed in models of suramin-induced preeclampsia [132], maternal exposure to L-NAME [133], and maternal nicotine exposure [134].

### 6.5. Others

There are other amino-acid-targeted interventions by which CKM phenotypes could be prohibited in adult progeny, such as supplementation with glycine [135], BCAAs [136,137], and tryptophan [138]. One study uncovered that perinatal glycine supplementation shields the offspring from hypertension induced by maternal low-protein intake [135]. This underscores the potential benefits of glycine in addressing human disorders, given its role in glutathione synthesis [158]. In a distinct approach, BCAA supplementation during pregnancy demonstrated efficacy in preventing hypertension primed by maternal caloric restriction in adult offspring [136]. Additionally, another study showcased the benefits of perinatal leucine supplementation in mitigating obesity and glucose intolerance in adult mouse offspring exposed to a high-fat diet during the perinatal period [137]. The inconclusive nature of previous studies addressing the association of BCAAs with hypertension [159,160,161] highlights the need for further investigations to comprehensively understand the reprogramming effects of perinatal BCAA use, especially in the context of hypertension. Thirdly, the reprogramming effects of perinatal tryptophan supplementation were assessed in a maternal CKD model [134]. The protective influence of tryptophan supplementation against hypertension in offspring, previously predisposed by maternal CKD, is associated with modifications in various tryptophan-metabolizing microbes and the AHR signaling pathway. While the inclusion of maternal methionine supplementation in a methyl-donor diet has demonstrated advantages for the later health of offspring [162], we opted to not include methionine in the list presented in Table 1. This decision stems from the recognition that its protective effects may be attributed to other nutrients involved in the one-carbon cycle metabolism.

It is noteworthy that the protective effects of various amino acids against CKM syndrome in offspring are intricately tied to changes in gut microbiota compositions. The chemical diversity of amino acids gives rise to numerous microbial metabolites with wide-ranging bioactivities, potentially mediating their prebiotic properties. While some studies have demonstrated synergistic effects of co-administering amino acids with probiotic bacteria to enhance human and animal health [157], uncertainties persist regarding their combined use in the DOHaD research field.

## 7. Conclusions and Perspectives

This review provides a thorough examination of amino acid effects during pregnancy on offspring outcomes, focusing on CKM syndrome. It consolidates existing knowledge and reveals new avenues for CKM syndrome prevention through targeted amino acid interventions. Supplementation with arginine, citrulline, taurine, cysteine, glycine, BCAAs, and tryptophan during pregnancy and/or lactation showed positive effects on CKM phenotypes in various animal models. While the association between dietary amino acids and gut microbiota is acknowledged, the precise mechanisms remain elusive, due to diverse biological activities.

Based on this review, we propose recommendations for future research. We advocate for improved study designs with better control, suitable animal models, standardized dosing, and optimal timing for amino acid supplementation. Understanding the distinct roles of individual amino acids and their interactions in CKM syndrome’s developmental programming is crucial. Future research should explore the optimal combinations of amino acids with potential probiotics or prebiotics.

There is limited information on translating reprogramming strategies from animal studies to pregnant women. Therefore, bridging the gap between human and animal research, particularly focusing on amino acid reprogramming strategies, is essential. Addressing these questions is critical, as early-life amino acid supplementation may provide novel therapeutic opportunities to reduce the global burden of CKM syndrome.

## Figures and Tables

**Table 1:** Perinatal amino acid supplementation to prevent cardiovascular–kidney–metabolic (CKM) phenotypes.

Amino acid Supplementation Dose	Period	Experimental Model	Species	Age at Evaluation (Weeks)	Protective Effects	Ref.
Arginine						
200 mg/kg/day	Lactation	Maternal protein restriction	SD rat/M	8	Hepatic insulin signaling and gene expression were prevented	[116]
Citrulline						
2.5 g/L in drinking water	Pregnancy and lactation	Maternal caloric restriction	SD rat/M	12	Kidney disease was prevented	[117]
2.5 g/L in drinking water	Pregnancy and lactation	Antenatal dexamethasone exposure	SD rat/M	16	Hypertension was prevented	[118]
2.5 g/L in drinking water	Pregnancy and lactation	STZ-induced diabetes	SD rat/M	12	Hypertension and kidney disease were prevented	[119]
2.5 g/L in drinking water	Pregnancy and lactation	Maternal L-NAME exposure	SD rat/M	12	Hypertension was prevented	[120]
2.5 g/L in drinking water	Pregnancy and lactation	Maternal CKD	SD rat/M	12	Hypertension was prevented	[121]
2.5 g/L in drinking water	From gestational day 7 to postnatal week 6	Genetic hypertension model	SHR/M and F	50	Hypertension was prevented	[62]
Taurine						
1.5% in drinking water	Pregnancy and lactation	Maternal high-fat/high-fructose diet	Wistar rat/M and F	21	Obesity was prevented in M	[122]
2.5% in drinking water	Pregnancy and lactation	Genetic hypertension model	NOD mice/M	50	Onset time of diabetes was postponed	[123]
3% in drinking water	Pregnancy and lactation	Maternal high-sugar diet	SD rat/F	8	Hypertension was prevented	[124]
3% in drinking water	Pregnancy and lactation	Maternal CKD	SD rat/M	12	Hypertension and renal hypertrophy were prevented	[125]
3% in drinking water	Pregnancy and lactation	STZ-induced diabetes	Wistar rat/M and F	16	Hypertension was prevented	[126]
3% in drinking water	Pregnancy and lactation	Maternal dyslipidemia	Wistar rat/M and F	16	Obesity, dyslipidemia, and hypertension were ameliorated	[127]
3% in drinking water	Pregnancy and lactation	Genetic hypertension model	SHR/M	22	Hypertension was prevented and diabetic retinopathy was attenuated	[128]
5% in drinking water	Pregnancy and lactation	Genetic hypertension model	SHRSP/M	12	Hypertension was prevented	[129]
Cysteine						
L- or D-cysteine, 8 mmol/kg/day	Pregnancy	Maternal CKD	SD rat/M	12	Hypertension was prevented	[130]
NAC, 1% in drinking water	Pregnancy and lactation	Prenatal dexamethasone and postnatal high-fat diet	SD rat/M	12	Hypertension was prevented	[131]
NAC, 1% in drinking water	Pregnancy and lactation	Suramin-induced preeclampsia	SD rat/M	12	Hypertension was prevented	[132]
NAC, 1% in drinking water	Pregnancy and lactation	Maternal L-NAME exposure	SD rat/M	12	Hypertension was prevented	[133]
NAC, 500 mg/kg/day in drinking water	From gestational day 4 to postnatal day 10	Maternal nicotine exposure	SD rat/M	32	Hypertension was prevented	[134]
Glycine						
3% in chow	Pregnancy and lactation	Maternal protein restriction	Wistar/M	4	Hypertension was prevented	[135]
BCAAs						
BCAA-supplemented diets	Pregnancy	Maternal caloric restriction	SD rat/M	16	Hypertension was prevented	[136]
1.5% in chow	Pregnancy and lactation	Maternal and post-weaning high-fat diet	C57BL/6 mice/M	16	Obesity and glucose intolerance were alleviated	[137]
Tryptophan						
200 mg/kg/day	Pregnancy	Maternal CKD	SD rat/M	12	Hypertension was prevented	[138]

SD, Sprague–Dawley rat; SHR, spontaneously hypertensive rat; SHRSP, stroke-prone spontaneously hypertensive rat; NOD, Non-obese diabetic; M, male; F, female; L-NAME, N^G^-nitro–L-arginine methyl ester; STZ, streptozotocin; CKD, chronic kidney disease; BCAA, branched-chain amino acid; NAC, *N*-acetylcysteine.

## Data Availability

Data are contained within the article.
